# Leveraging conscious and nonconscious learning for efficient AI

**DOI:** 10.3389/fncom.2023.1090126

**Published:** 2023-03-23

**Authors:** Rachel St. Clair, L. Andrew Coward, Susan Schneider

**Affiliations:** ^1^Simuli Inc., Delray Beach, FL, United States; ^2^College of Engineering and Computer Science, Australian National University, Canberra, ACT, Australia; ^3^Center for Future Mind, College of Arts and Letters, Florida Atlantic University, Boca Raton, FL, United States

**Keywords:** the recommendation architecture, deep learning, consciousness, novelty, feedback, resource constraints, artificial intelligence

## Abstract

Various interpretations of the literature detailing the neural basis of learning have in part led to disagreements concerning how consciousness arises. Further, artificial learning model design has suffered in replicating intelligence as it occurs in the human brain. Here, we present a novel learning model, which we term the “Recommendation Architecture (RA) Model” from prior theoretical works proposed by Coward, using a dual-learning approach featuring both consequence feedback and non-consequence feedback. The RA model is tested on a categorical learning task where no two inputs are the same throughout training and/or testing. We compare this to three consequence feedback only models based on backpropagation and reinforcement learning. Results indicate that the RA model learns novelty more efficiently and can accurately return to prior learning after new learning with less computational resources expenditure. The final results of the study show that consequence feedback as interpretation, not creation, of cortical activity creates a learning style more similar to human learning in terms of resource efficiency. Stable information meanings underlie conscious experiences. The work provided here attempts to link the neural basis of nonconscious and conscious learning while providing early results for a learning protocol more similar to human brains than is currently available.

## 1. Introduction

Philosophy and neuroscience have long sought to understand the basis of consciousness (Velmans and Schneider, 2007; Seth and Bayne, 2022). There are several leading theories, none of which has been sufficiently to become the standard working theory of the subject. As a result, several interpretations of the neuroscience behind cognitive phenomena, such as learning, have deliberately not presupposed a particular theory of consciousness (Godfrey-Smith, 2021).

In this paper, we explore how the type of information termed “consequence feedback” could be used by the brain and how different uses of consequence feedback change the type of information that can be activated in consciousness. In consequence feedback, an action taken by the brain affects the environment, which in turn affects the brain. Consequence feedback can contain different amounts of information. In the lowest information case, the environment only provides an indication of whether the behavior was correct or incorrect, with no additional information about the nature of the correct behavior. In a higher information case, the correct behavior is specifically indicated by the environment, *via* some kind of teacher. An even higher information case is when the difference between the actual behavior and target behavior (an error signal) is provided. Consequence feedback can be conscious or nonconscious.

In conscious consequence feedback, the brain can use multiple mechanisms like working memory, attention, agency, sentience, etc. to interpret the signal more or less strongly. One type of conscious consequence feedback is a teacher signal. In this type of feedback there is a direct awareness of the relationship between the agent's actions and the resulting reaction of the environment back onto the agent; an obvious corrective signal is received following behavior. Teacher signals can be either of the two types of consequence feedback (low or high information). As it is often the case in child development, teachers can provide the correct answer by example and by corrections to mistakes, or can provide rewards (positive or negative) for responses and behavior.

In nonconscious consequence feedback, the brain may also use multiple mechanisms like working memory, attention, agency, sentience, etc.. However, the distinction here is that the agent may not be able to report the awareness of the external signal. Blindsight and visual masking are both examples of nonconscious consequence feedback, although there are many such examples Lewicki et al. (1992), Phaf and Wolters (1997), Olcese et al. (2018), and Birch et al. (2020).

Alternatively, non-consequence-feedback information mechanisms do not rely on external environment feedback and are far more likely to be nonconscious events. In this type of learning, reward or error signals may still be occurring in the form of associated consequence feedback neurochemicals (e.g., dopamine) (Papalini et al., 2020). The important difference is that these alternatives do not entirely rely on a feedback signal, but can instead rely on other learning mechanisms, such as plasticity with BDNF neurochemicals, Hebbian learning and spike-timing-dependent-plasticity in cortical pyramidal neurons in Kowiański et al. (2018) and Coward (2021).

There are several interpretations of the role consequence feedback plays in learning and consciousness. In Global Workspace Theory, proposed by Baars, et al., consciousness arises from a shared latent space in the brain composed of signals broadcast from specialized modules, which relies on a type of error termed cycle-consistency (VanRullen and Kanai, 2021). In Predictive Processing proposed by Karl Friston et al., the error between an expected event and actual event is predicted and these predictions are basic computations throughout the brain that drive learning, which in part create access consciousness (Friston and Kiebel, 2009; Marvan and Havĺık, 2021). Integrated Information Theory, proposed by Tononi et al. does not provide a clear relationship between learning mechanisms, or consequence feedback information, and consciousness (Tononi et al., 2016).

According to the Recommendation Architecture (RA) proposed by Coward, consequence feedback is a brain mechanism used to learn how to interpret cortical patterns of activation (Coward, 1999a). The interpretations take place in subcortical structures, and the patterns of cortical activation themselves have alternative learning protocols based on temporal correlations and are not dependent on consequence feedback. These mechanisms (along with others) work together to promote different degrees of consciousness.

In the RA, consciousness arises as a result of indirect activation of cortical neurons in the absence of the sensory inputs that would activate them directly (St. Clair, 2020). Such indirect activations are the result of interpretation by subcortical structures of the preceding pattern of cortical activation. The subcortical interpretations are driven by consequence feedback. Because the circumstances in which cortical neurons are activated are not directly changed by consequence feedback, indirect activations can be very similar to direct activations in response to sensory inputs. As a result, there are significant similarities between sensory experiences and imagined experiences (Coward and Gedeon, 2009; Coward, 2013). We do not implement indirect activation in this work, but our contributions serve to lay the groundwork for future instantiations and for better understanding the RA framework.

Thus, it's important to better understand how consequence feedback affects learning and specifically, to what extent it drives new learning. Further, identifying how environmental signals influence learning informs our understanding of how cognitive phenomena like attention, working memory, agency, etc. are utilized in conscious activity.

In the field of artificial intelligence (AI), state-of-the-art artificial learning models fail to embody the intricacies of the brain's learning process. Most approaches employ teacher signal consequence feedback as the driving and often sole learning force.

The main algorithm dominating the AI field is backpropagation, which is used in nearly all popular deep learning approaches (a subfield of AI). In backpropagation, an error signal is mathematically propagated from the final output layers of the network to the first layers, such that each neuron model receives direction on how useful its representation was in completing a task correctly (Chauvin and Rumelhart, 2013). The plausibility of backpropagation as an actual neural mechanism continues to be debated (Whittington and Bogacz, 2019; Lillicrap et al., 2020).

Alternatively, in another subfield of AI, reinforcement learning (RL), a reward (positive or negative) is given to each state-action pair (behavior given an input stimuli) and the learning algorithm is primarily based on maximizing expected reward (Sutton and Barto, 2018). The amount of reward is either pre-determined from a teacher signal or a function of the teacher signal.

These methods have shown to be very effective for narrow domain learning in which one specific task is learned in a repetitive process with the goal to extend this learning to unseen examples within the same task and data domain. With few exceptions, there are two glaring departures between how the human brain learns and how AI models learn. First, learning a task without excessive repetitive memorization of the input stimuli, i.e., learning novelty efficiently. Secondly, the ability to learn something new and return to previous learning without exponentially increasing resources required to learn or catastrophically diminishing performance, i.e., retaining prior learning. Both of which the brain can do, but AI has not been entirely successful at.

For example, the popular AI model, Long Short-Term Memory (LSTM) as introduced in Hochreiter and Schmidhuber (1997) is capable of learning to trade financial stocks and is also the backbone of speech recognition in Apple's Siri (Levy, 2016; Roondiwala et al., 2017). However, each instance of the model is limited to the task it was trained to perform. For example, if an LSTM model has been trained to understand speech, it cannot then be used to trade stocks. If you retrain that same model to trade stocks, it cannot then understand speech. Current AI models struggle in returning to a previously learned task without re-learning that task.

Furthermore, to learn any task, AI models must iterate over the same dataset hundreds and sometimes thousands of times. While there is much structure in the natural world, no two sensory inputs are ever identical. The way photons hit your retina is never quite the same as the moment before. In the natural world, it is difficult to experience the same input condition more than once.

The overall issue with these approaches is that they lack the ability to scale efficiently. More computational resources are needed continuously as the model learns. The human brain is able to learn within resource constraints. Some work in the RA describes how learning within resource constraints is an important feature of generalizing (Coward and Gedeon, 2009). Specifically, that consequence feedback cannot be used as the sole learning mechanism because it would require constant addition of new resources (i.e., neurons).

Thus, current state-of-the-art AI suffers from an inability to learn novelty while retaining prior learning within resource constraints, known as narrow learning.

While consequence feedback driven models have been successful at some form of learning, the lack of learning novelty efficiently while retaining prior learning, suggests that these current models learn quite differently than their biological counterparts. This discrepancy, amongst others, make them poor models for studying cognition and consciousness as it occurs in humans.

From the perspective of cognitive science, we can formulate the problem as whether learning only occurs through external signals. Here, we argue consequence feedback is an externally driven activity and is only one of the several mechanisms driving learning.

An agent makes a decision which impacts its environment. The environment or another agent then provides feedback on how that decision affects the agent. Consequence feedback may be conscious (e.g., teacher signals) or nonconscious (i.e., blindsight). As an alternative to consequence feedback, a large body of literature suggests there are forms of learning that do not directly involve a teacher signal or sensory feedback (Blakemore and Mitchell, 1973; Perruchet, 1994; Aarts et al., 2009; Coward, 2010; Reber, 2013). These forms may involve nonconscious states, such as dream sleep (Coward, 2009). Thus, the question then becomes what does non-consequence-feedback have to offer general learning?

From the perspective of AI, the question is whether restricting consequence feedback to portions of learning models and allowing for non-consequence-feedback learning algorithms in other portions affords more efficient novelty learning than completely consequence feedback driven learning models.

Here, novelty learning can be measured in part by the totality of processes involved in learning new stimuli more accurately or with fewer parameters and memory even in the event of returning to prior tasks after learning new tasks.

### 1.1. The recommendation architecture

Since its initial proposal in Coward (1990), the recommendation architecture has been used to understand the roles of different brain anatomical structures in considerable detail (Coward and Coward, 2013), and has been electronically simulated to some degree (Gedeon et al., 1999; Coward, 2001, 2004b). The fundamental property of the RA is that there is a sharp separation between a subsystem (called clustering) that defines and detects conditions in the information available to the brain and a subsystem (called competition) that interprets a condition detection as a range of recommendations in favor of different behaviors and selects and implements the most strongly recommended behavior. Consequence feedback changes the recommendation weights in the competition subsystem that resulted in recently selected behaviors, but cannot directly affect the condition definitions themselves. Condition definitions can only be changed on the basis of temporal correlations, and the circumstances in which changes can occur are severely restricted. The reason for such restrictions is that any such changes can affect the integrity of all the other behaviors recommended by the changed condition. In particular, using consequence feedback to change a condition definition might improve the definition for a recently selected behavior, but would generally make the definition less applicable for all the other behaviors it also recommends. Even on the basis of temporal correlation, condition changes are most often limited to slight expansions in the range of circumstances in which the condition is detected. The simplest conditions are initially defined by combinations of sensory inputs that often occur at the same time. Higher level conditions are defined by combinations of simpler conditions that often occur at the same time. Different modules in the clustering subsystem define and detect conditions on different levels of complexity that are effective for recommending different types of behavior. If every sensory input and detected condition needed to be processed, the resources required would be excessive. Subsets of inputs and detected conditions are therefore selected for processing. Such selections are behaviors that are recommended by condition detections.

In the brain (Coward and Coward, 2013), the cortex corresponds with the clustering subsystem, and different cortical areas correspond with modules detecting conditions on different levels of complexity. The hippocampus determines when condition definition changes are appropriate in the cortex and implements the changes. The basal ganglia correspond with the competition subsystem. The thalamus implements any behaviors of releasing information into or between cortical areas once those behaviors have been selected by the basal ganglia on the basis of its cortical inputs. The amygdala and hypothalamus detect conditions that influence the selection of different general types of behavior. Selection of behavior by the basal ganglia takes a certain amount of time. Some behaviors are often used in exactly the same sequence. Examples include the sequences of muscle movements used fro walking, climbing stairs, or generating frequently used words and phrases. Implementation of such sequences can be speeded up by recording the sequence and limiting the basal ganglia to selection the sequence once as a whole rather than selecting all the individual muscle movements. The cerebellum records and implements such behavior sequences.

Conditions can be directly activated by a re-occurence of the sensory circumstances in which they were defined. A condition can also be indirectly activated on the basis of the activity of other conditions that were active at the same time as its past activity. These indirect activations are also behaviors which must be recommended by current condition detections and accepted by the basal ganglia. Indirect activations are the basis for declarative memory, speech and consciousness (Coward, 2010). Because of the severe restrictions on the type of changes to condition definitions, such indirect activations have significant subjective similarities to direct sensory experiences.

There is a fundamental difference between the recommendation architecture and cognitive architectures like SOAR or ACT-R. In all types of architecture, the system is separated into components (which may be called subsystems or modules). In SOAR (Laird, 2022) or ACT-R (Ritter et al., 2019), these components correspond with major cognitive processes like visual perception, procedural memory, semantic memory, episodic memory etc. In the recommendation architecture, the driving force defining the separation into components is the need to minimize the total information processing resources required. Each component is a chunk of information processing resources optimized to perform one type of information process very efficiently. A cognitive process is carried out using information processes performed by many if not all components. In a sense, models like SOAR and ACT-R are analogous with a user manual for a computer, in which the major components are applications performing different user functions. The recommendation architecture is analogous with the system architecture of a computer, in which the major components are CPU, memory, WI-FI interface, monitor driver etc. and any application requires information processes performed by most or all of the components. It would be possible to implement a computer in which different physical modules corresponding with different applications, but such an implementation would be very costly in terms of total resources needed. In an architecture in which resources are a significant constraint, there will be no direct correspondences between components and user features.

### 1.2. Novel contributions

We consider a categorical learning task where the objective of the model is to correctly identify an unseen stimuli's category. No two stimuli are ever the same throughout training and testing. Three models are evaluated. First a novel model, which we refer to as the RA model, simulates a very basic cortex, basal ganglia, thalamus, and hippocampus. The cortex uses an alternative learning mechanism where conditions are defined by identifying combinations of information that often occur simultaneously during experience. In this model, consequence feedback is only used on the basal ganglia. We then compared two state-of-the-art (SOTA) AI models: DQN and ResNet (Van Seijen et al., 2009; Mnih et al., 2015; He et al., 2016). More on each model is given in the background and approach sections. The results on the task are collected in terms of accuracy, number of nodes, number of learning interactions, and random-access-memory – RAM (as an indication of computational steps).

We expect the RA model to outperform the SOTA AI models on the categorical task because it's quicker to learn novel stimuli. If the RA model uses consequence feedback to inform the accuracy of behavioral interpretations of condition definitions created in the cortex, the ability of the model to combine representations of information is extended without having to extensively update values of nodes representing such information.

The key insight here is that entirely consequence feedback driven learning interferes with the system's ability to learn novel conditions because such models must rewrite each node (i.e., neuron) to define only the current input condition. Nodes cannot easily be used in the instance of a new stimuli since they define a specific condition in the last set of inputs. This problem is known as catastrophic forgetting, in which prior learning is rewritten for the sake of current learning (Goodfellow et al., 2013).

In contrast, a condition definition driven learning system, as described in the RA, may be more akin to human learning. RA literature suggests consequence feedback is used to interpret cortical activity rather than create the activity itself (Coward, 1999a). Thus, error or reward from external stimuli cannot be used to directly change cortical activations. Instead consequence feedback is used to learn to interpret cortical activity.

Ultimately, the benefit of such an approach is faster learning of novel conditions without excessive resources. In our model, this manifests as the ability to combine cortical representations in a more expressive manner, which defines novel stimuli conditions quickly without degrading their use for other stimuli condition definitions.

We further explore this claim by investigating each models' performance on returning to prior learning after new learning. The final results of the study show that consequence feedback as interpretation, not creation, of cortical activity creates a learning style more similar to human learning in terms of resource efficiency.

## 2. Background

### 2.1. Consequence feedback in artificial intelligence

In AI, the most widely known and arguably best performing models (such as GPT-3, Gato, AlphaGo, DALL-E, etc.) are those which use some form of backpropagation or reinforcement learning as the driving learning algorithm (Silver et al., 2016; Gundersen and Kjensmo, 2018; Brown et al., 2020; Zhang and Lu, 2021; Reed et al., 2022; Tewel et al., 2022). Backpropagation is a type of consequence feedback since an error signal derived between the current behavior and desired behavior is propagated mathematically to each node in the network. Using a partial derivative equation, the error signal provides a measure for how much to change the value of each node to elicit a more accurate response during the next round of stimulus (Chauvin and Rumelhart, 2013). Reinforcement learning (RL) uses an adaptation from Markov decision modeling to predict the reward expected when performing a behavioral output, given a particular input (Sutton and Barto, 2018). From many previously performed and saved input-behavior (i.e., state-action) pairs, the goal in RL is to learn or use a function which maximizes reward of a new state, thereby learning the correct action. Both backpropagation and reinforcement learning can be thought of as consequence feedback mechanisms.

ResNet is a deep neural network that excels at learning categorical data (He et al., 2016). Resnet uses backpropagation as the sole learning algorithm. Deep-Q Network (DQN) is a deep reinforcement learning neural network that shows promising results in reward-driven learning tasks (Mnih et al., 2013). DQN model is a mixture of backpropagation and reinforcement learning.

A common problem in backpropagation neural networks is catastrophic forgetting (Goodfellow et al., 2013; Clair, 2022). Usually, during the backpropagation algorithm, the signal that informs how to change each node is given only by an error signal from the most recent set of inputs. The nodes are updated according to the assumption that future inputs are similar to current inputs. Thus, when a task changes, from one domain of data to another, the nodes have to begin the updating process again, in repetition until the nodes have changed significantly to define the new set of inputs; the model has forgotten (catastrophically) previous knowledge.

To our knowledge, there is no known solution for completely overcoming catastrophic forgetting. There have been some attempts to assuage the problem of catastrophic forgetting with delayed backpropagation, entropy based backpropagation, tokenization of networks, etc. (Kirkpatrick et al., 2017; Shmelkov et al., 2017; Serra et al., 2018). Promising efforts recently introduced in SEER show an alternative self-supervised learning style that can process orders of magnitude more data than traditional neural networks (Goyal et al., 2021). In SEER, prediction errors between unseen or masked data and the reveal of what the masked data contains, drives learning through a process termed contrastive learning.

Although all of these approaches are helpful, they do not actually reduce the fundamental problem of needing to increase computations to increase learning capacity; eventually the capacity of the system is reached without fundamentally re-designing the learning algorithm itself. This approach is quite antithetical to the way the brain learns. To give perspective on how critical resource efficiency is, one recent SOTA natural language model, GPT-3, cost $4.6M to train (Li, 2022).

Likewise, in RL a similar resource expenditure problem is observed. By design, RL models store a record of state-action-reward tuples. As learning occurs over time, more states, actions, and resulting rewards are recorded. This leads to excessive computational memory as the model scales and limits the lifetime of the model within reasonable costs of computation. A single training regimen for one of the most prominent RL deep neural networks, Gato, costs roughly $50k to learn to navigate a few simulated control environments (Effective, 2022).

Some approaches have been proposed to assuage the scaling issues within RL. One such work, exploits multiple processing units that compute locally before pushing computations to a shared repository in such a way that convergence to a solution scales linearly (when compared to previous methods) (Zhan et al., 2017). Similarly SeedRL achieves some efficiency improvements by leveraging a distributed cluster of software and hardware components which are then connected to a central inference area *via* a fast streaming technique (Espeholt et al., 2019).

Thus, the general approach to being able to increase the knowledge repertoire in AI seems to be to increase the number of nodes or disperse the computations, rather than actually reduce the number of computations and memory requirements needed to learn more information. This trend results in a failure of resource efficiency and is one of the reasons neural networks are so expensive to train.

Yet, there remains the problem of being able to learn novelty efficiently as is seen in the human brain. In AI a technique known as few-shot learning has shown some ability to learn novelty quickly (Wang et al., 2020). Traditionally in few-shot learning, a limited number of training inputs and corresponding labels are given, the model learns on this subset before performing on a much larger subset. This method is the inverse of traditional AI where the training inputs are much larger than the performing inputs (i.e., test inputs). Techniques proposed in these works rely on consequence feedback since the error signal or reward is still given in the smaller training subset in the form of labels.

There are variations to this technique, namely one-shot learning, zero-shot learning, and meta-learning (Wang et al., 2020). In one-shot learning, the training subset is reduced to only one example of each category in the task. In zero-shot, no examples from the task are given, but instead a large amount of other data is provided. Meta-learning augments a traditional learning approach with a secondary learning mechanism as a function of the first learning mechanism. This second learning mechanism is typically a variation of zero-shot learning. With the exception of zero-shot learning, these approaches utilize consequence feedback to drive learning. Zero-shot learning typically doesn't use any consequence feedback.

The RA model proposed can be thought of as a type of few-shot learning, but does not only augment existing learning protocols, it is itself a new learning protocol. The model uses a combination of non-consequence-feedback and consequence feedback to drive learning. The focus of this work is on the effects of consequence feedback in learning novelty and retaining knowledge under resource constraints rather than the ability to learn from as few examples as possible (i.e., few-shot learning).

### 2.2. Consequence feedback in the brain

Traditionally, consequence feedback and the role of explicit reward has been the primary target for understanding learning mechanisms in the brain. However, there is an alternative understanding of anatomy and physiology that describes the process of learning and role of consequence feedback. In the theory proposed by Andrew Coward, the Recommendation Architecture (RA), consequence feedback is described as a tool to learn how to interpret cortical patterns of activation, while the patterns of activation have an alternative learning protocol not dependent on consequence feedback (Coward, 2001).

In RA, the cortex defines conditions by identifying combinations of information that often occur together in experience (Coward, 2021). The basal ganglia associates the final output of the cortex with a range of recommendations in favor of different behaviors, and implements the behavior with the largest total recommendation strength across current cortical outputs.

Initially, recommendation strengths are assigned on the basis of guidance, the correct behavior is indicated by a teacher. Subsequently, simple correct/incorrect consequence feedback reward is provided following the selection of a behavior. Incorrect feedback decreases the recommendation weights that were active in recommending recent behaviors. Conditions detected within the circumstances following the behavior may have recommendation strengths in favor of decreasing the recommendation weights that were active in recommending slightly earlier behaviors. Correct feedback is used less often to increase such recommendation weights.

In order to create an adequate range of recommendations, at least a minimum number of conditions must be detected in every situation. If fewer than this minimum number of conditions are detected (i.e., if there is some degree of novelty in the current situation), some undetected conditions expand so that they also are detected and add their recommendation strengths. Furthermore, signals indicating strong emotion can increase the number of expansions. However, condition expansions performed to achieve the minimum number can decay over time, and need to be confirmed by signals indicating that the expansions are worth keeping. Consequence feedback cannot guide the selection of what condition changes take place, but can confirm that changes driven by novelty should be retained in the long term. Positive and negative consequences have essentially the same effect on retaining condition changes.

**Figure 1:**
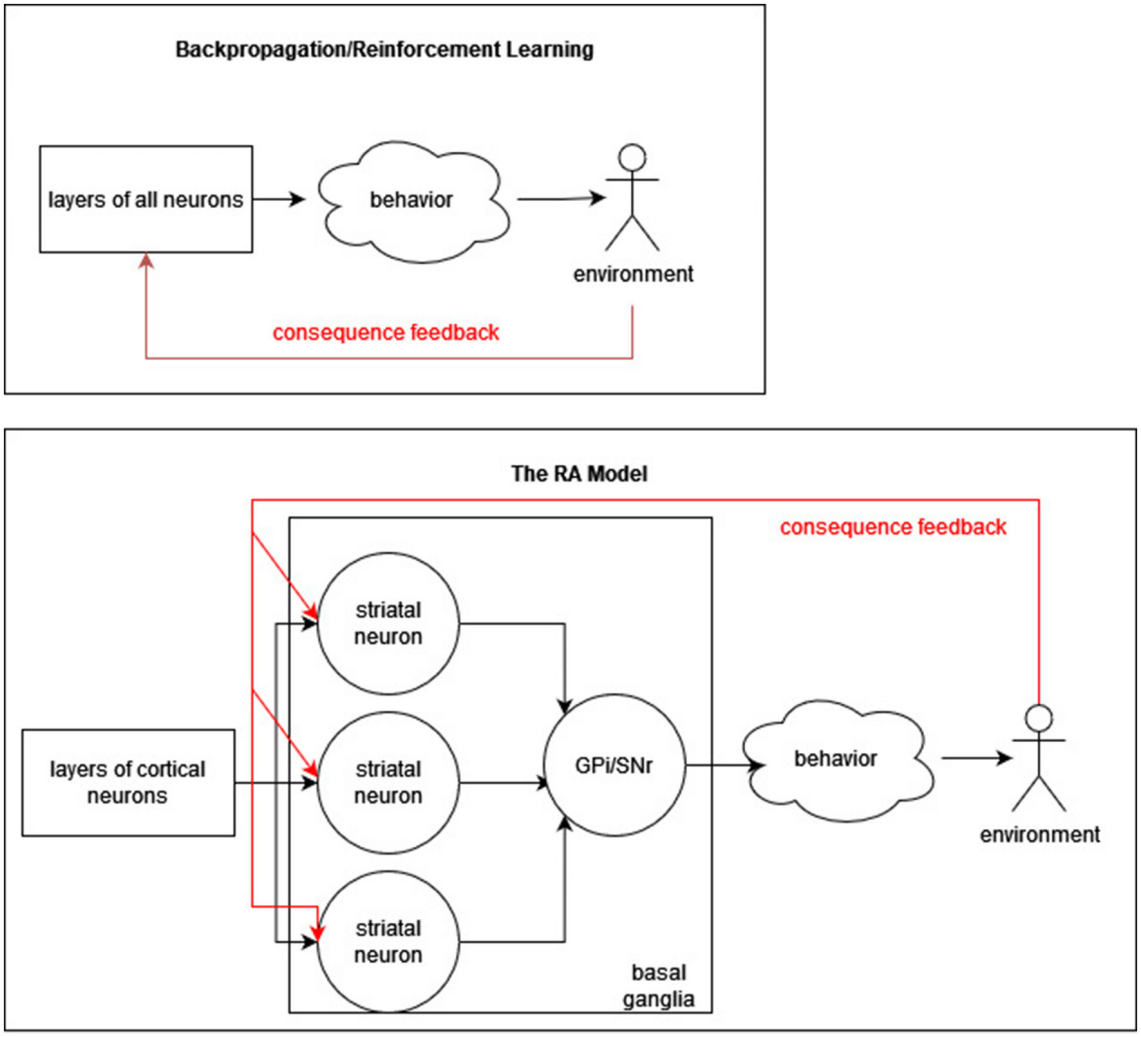
Recommendation architecture vs. backpropagation. A simplified diagram of the difference between RA **(bottom)** and backpropagation/reinforcement learning **(top)** is presented. In RA, consequence feedback does not modify cortical neurons, whereas it does in error and reward models.

The cortex identifies conditions that tend to occur relatively often in experience, in such a way that two conditions tend to be different (i.e., do not always occur at the same time). If a new behavior needs to be learned and if past experience has included circumstances in which the new behavior is appropriate, conditions appropriate for recommending the new behavior may already exist, just requiring the assignment of recommendation weights. Thus, learning a new behavior may be somewhat easier. Figure 1 depicts the difference in use of consequence feedback (red arrows) between the RA model and backpropagation/reinforcement learning models.

Learning new behavior more efficiently can be measured in part by accuracy. It's also important to take into account the total scope of learning, which includes the total training size, the number of nodes needed, the hardware memory utilized, number of operations performed, etc..

Thus, we aim to determine how the accuracy of learning categories relates to the amount of nodes in the network learned and random access memory (RAM) used by the network. Number of nodes and number of learning iterations were chosen as basic computational resources being utilized since it's readily available and comparable between models. Since the RA model is written in the programming language Smalltalk and the SOTA AI models are written in Python, it is difficult to extract the number of operations, memory, and speed in a comparable fashion. However, RAM is reported as an approximate performance metric although a direct comparison should be taken lightly since models are not run on the same hardware and language frameworks. Benefits in learning with fewer resources have shown to be promising tools in emerging technologies and to alleviate the drawbacks of costly neural networks (Hernandez, 2020). Furthermore, learning novelty efficiently may provide a more realistic model of human intelligence than is currently available.

## 3. Approach

**Figure 2:**
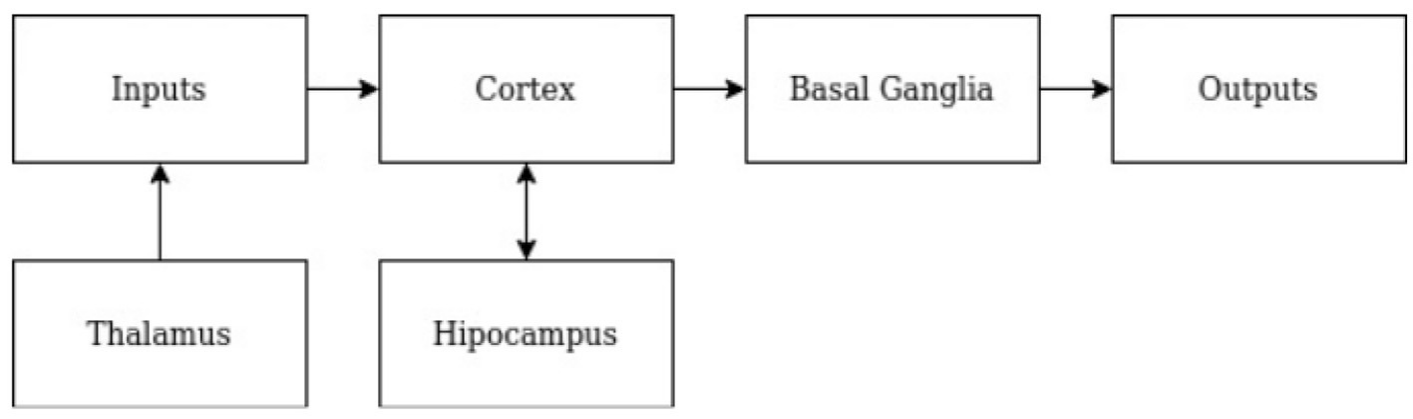
RA model overview. A block diagram depicting components of the RA model and the direction in which information flows.

Traditional SOTA techniques in AI utilize consequence feedback as the driving learning mechanism, either in the form of error, reward, or both. These methods are unlike human learning because they fail to learn novelty efficiently and retain prior learning after new learning within a constrained resource environment. These methods require many iterations and copious amounts of computation. Learning is impeded by the need to constantly re-learn the nodes' representation of the current task, slowing the learning process. We present a model of RA that accurately learns novelty efficiently, per number of nodes, learning iterations, and memory. The model is constructed according to prior instantiations of RA in Gedeon et al. (1999) and Coward (2004a,b). In the RA model, there are four components: cortex, basal ganglia, hippocampus, and thalamus. Figure 2 depicts the flow of information through each module in the RA model.

### 3.1. The RA model

#### 3.1.1. Cortical component

The simulated cortex has three layers of neurons, organized into columns. In each column there are 10 layer one neurons, 10 layer two neurons, and one layer three neuron. Each neuron has multiple dendritic branches (approximately 1,200 synaptic connections) and each branch has multiple inputs from the preceding layer or from external inputs in the case of the first layer. Interlayer connectivity is within columns. Neurons produce spike inputs if an integration process within each of its branches followed by an integration process across branches exceeds a threshold. For simulation purposes a time slot is defined as a (conceptual) third of a millisecond, and the neurons are updated once every timeslot.

#### 3.1.2. Basal ganglia component

There is only one layer three neuron in each column, and each such neuron targets the basal ganglia. The simulation of the basal ganglia is very simple. There is one basal ganglia conceptual neuron corresponding with each of the 30 categories, and each layer three cortical neuron has a recommendation weight in favor of each behavior. These weights all start at the same low value, and increase or decrease in response to reward feedback. There are two types of reward: teacher signal, where the correct category is identified, and consequence where the feedback is just correct/incorrect. Rewards are not used in the cortex. As a result, after one cortex run is completed it is possible to experiment with different basal ganglia parameters.

There is one basal ganglia neuron for every category. Layer three outputs go to these basal ganglia neurons. Each such layer three output has a range of different recommendation weights in the basal ganglia in favor of different categories (i.e., of identifying the current input as a member of a category). The basal ganglia identifies the category of the current input by adding the recommendation weights of all the layer three outputs over the 200 ms period in favor of each category, and selecting the category with the largest total. This means that 2nd, 3rd, etc. choices can also be identified.

During training, the basal ganglia is told the correct identification, and uses this to adjust the recommendation weights. The system is then tested by exposure to a new set of input states, and the results calculated. The result is the category with the highest total recommendation weight, but 2nd and 3rd etc. choices are also calculated. In addition, the basal ganglia can be given just the information that its selection is correct or incorrect. When a new set of categories are learned, the new learning can partially interfere with prior learning. The existence of information on 2nd and 3rd etc. choices means that the prior learning can be largely restored using just incorrect feedback without information on the actual identity of the correct category.

#### 3.1.3. Thalamic component

The function of the thalamus is modeled by 40 Hz frequency modulation of the spikes, which also makes it possible to present three different categories in each 200 ms period (modeling attention/working memory to a degree). This modulation occurs on the input categories before the model begins learning. A behavior decision is made for each category in the working memory separately, because modulation separates the categories in time.

#### 3.1.4. Hippocampal component

The Hippocampal component drives learning in the cortical component, without consequence feedback.

Cortical neurons have branches and each branch has a number of synapses made by different inputs. Inputs to branches are integrated separately, and then the branch outputs are integrated to determine if the neuron fires. Neuron connectivity is initially defined randomly in the cortical component, but for each column there is a bias in favor of a different group of inputs that have tended to occur at the same time across presentations of all categories. This bias corresponds with dream sleep and is the only role of the hippocampus that is simulated. All inputs start with the same synaptic weight. From then on, a synaptic weight increases if there is an input to the neuron on that synapse just before the neuron fires. However, unless this sequence occurs at least three times within 200 ms, the increase is reversed. Furthermore, if a branch fires a number of times but each time some synapse does not contribute, the weight of that synapse is decreased. If a neuron fires very often, all the synaptic weights on the neuron are reduced by the same proportion.

### 3.2. SOTA AI models

To compare the novel model to traditional AI approaches, we test ResNet and DQN on the same learning task. The DQN was chosen as it represents both error and reward driven consequence feedback and has shown promising results in learning novelty amongst tasks. However, the RA model can also learn without receiving error or reward, only information that the category identification was incorrect. To compare, a reward only learning model is needed, which only gives a zero or one value indicating correct or incorrect, for which the model E-SARSA (Van Seijen et al., 2009) was chosen. However, we could not get the E-SARSA model to train without error based reward. Future results will attempt to reconcile only reward based learning with the results proposed here. To further understand the role of error and reward in consequence feedback, ResNet was chosen as a only error driven learning model which has shown SOTA success at category learning.

A hyperparameter tuning technique from Biewald (2020) was used for each SOTA AI model to assess the best model configurations for the task. Details of hyperparameter tuning and resulting hyperparameters for each model are provided in the Supplementary Sections 1–4. The important note is that during this tuning process, the ResNet and DQN were provided with the ability for more or less parameters and learning iterations. Both models required more than the lowest number of parameters and learning iterations to perform the task accurately. Thus, these models do require the number of computations listed above to achieve comparable accuracy to the RA model.

### 3.3. Experiments

In a series of experiments, we examined how consequence feedback can affect learning. A category classification task is used across all experiments. There are two general types of overall simulation runs. The first kind is to learn all 30 categories at once. This type is used to assess how efficiently and accurately novelty is learned. The second type is to learn only 15 categories, then another 15 categories and then accuracy of identifying all 30 categories is accessed. This type of experiment is used to understand how new learning interferes with prior learning.

In the RA model, each run type models about 5 min of cortex time, but actually takes many hours of simulation time. Multiple different instances of each category are presented, each presentation for 200 ms neuron time.

#### 3.3.1. Categorical task

Across experiments, the same categorical learning task is used. The task simulates a situation in which the cortex never experiences exactly the same input twice. An input vector of 400 components is presented. The components can either be zero or one. What determines if there is a zero or one in each component is a combination of the category, modulation, and chance. We can understand a one as indicating an input spike, a zero indicating no spike.

Each category is defined by a unique probability distribution which is determined at random at the start of each model instantiation. One category probability distribution is 400 components which are a number chosen at random from a list of numbers 0 to 200. Smaller numbers are repeated up to five times in the list and a decay occurs for the number of repetition as numbers increase in value. The point of this is to make spiking less likely since larger numbers increase spike likelihood.

The modulation factor is kept constant and pre-determined according to a 40 Hz frequency modulation pattern. The objective of this factor is to simulate the thalamus frequency modulation which makes working-memory possible in the RA model.

Chance (of a spike) is introduced by drawing a random number from a list from one to 10,000. If the product of the category probability for a specific component location and the modulation factor for a specific component location is greater than the random number, a spike occurs. If the product is less than the random number, a spike does not occur and instead a zero is placed as the component value at that location.

Size 400 was chosen for the inputs because in the RA simulation there are 400 input sources to the cortex (i.e., possible inputs to layer one neurons). The presentation of one object (one instance of a category) is a sequence of action potential spikes across the 400 component input. In the RA model, the presentation lasts for a notional 200 ms divided up into 1/3 ms time slots (for computational efficiency). One 400 component input stream is effectively one third of a millisecond, such that for 200 ms of presentation 600 of the 400 inputs are needed.

In the RA model, a type of working memory is utilized in which across the 600 presentations, three categories are presented one after the other. The thalamic module groups each category into approximately 8 ms for eight cycles representing the period of the 40 Hz gamma modulation. This means that 75 periods are needed to represent all three categories, each of 8 ms. Effectively, this results in a 200 ms presentation of synaptic inputs onto the first layer of cortical neurons. In the visual cortex, attention to an object lasts about 200 ms (Agam et al., 2010).

We've adopted the modulation approach in the SOTA AI models as well since it directly affects the distribution of spikes in the input. However, the SOTA AI models do not have the capacity for working memory. Some results on trying to show inputs with working memory (three categories at once) in the DQN model are reported in the Supplementary Section 5. As such, only one category is presented at a time. Eight cycles are still used but only 25 periods are needed to represent one category. The result is a 200 by 400 input presentation, which is approximately 67 ms of presentation time.

No presentations of two instances of a category will be the same, at least the probability that this occurs is extremely low due to chance. The objective is to re-create the inputs in a comparable way as they occur in the RA model. Effectively, the RA model is evaluating one category at a time, making it possible to compare SOTA AI models evaluating a different input size, corresponding to one category at a time modulated by thalamic frequency.

#### 3.3.2. Experiment one

In the first type of run, instances of all 30 categories are presented repeatedly in a sequence. The sequence of which the categories are presented never changes. Each model attempts to identify the corresponding category label (i.e., category one, category two… and so forth).

In the RA model, initially the output from every cortical column is given a connection on to the basal ganglia neuron corresponding with every category. All of these connections are given the same initial weight. These are the initial recommendation weights of the columns in favor of recognizing the categories. The cortex gets a sequence of 1,200 presentations of different category instances (40 of each category), each lasting 200 ms of neuron time. Cortical learning takes place, without consequence feedback, on the basis of temporal correlations: each column defines a group of conditions on the basis that conditions in the group tend to occur at similar times. For the first 300 presentations there is no use of the cortical outputs by the basal ganglia. In the next 600 iterations, if sufficient conditions in a cortical group are detected, the layer three cortical neuron output goes to the basal ganglia. The identity of the correct category is also communicated to the basal ganglia. In the basal ganglia, the total of (input spikes x recommendation weight) is calculated for each category. The category with the largest total recommendation weight is determined. Then the weights in favor of the correct category for each column are increased in proportion to the number of spikes generated during the presentation by the column. Finally, if the category with the largest total recommendation weight was incorrect, the recommendation weights in favor of that category for any columns that produced an output are reduced by a standard proportion. For a final 300 presentations, the predominant recommendation strengths are determined without consequence feedback, analogous to a test set in AI with the exception that the cortical nodes continuously update.

Here, the DQN and ResNet models are compared. Model training (i.e., learning) occurs over various iterations needed to reach similar accuracies of 77 and 90% accuracies. After the models are trained, another 300 iterations are used for testing.

The goal is to determine if the RA model has an advantage in learning novelty with less resource consumption. We expect the results to show that more memory and parameters are needed for the DQN and ResNet models with comparable accuracy on the task.

If we want to claim that condition definition (i.e., feature detection) which is not determined by consequence feedback can learn novelty with few resources, a similar accuracy has to be achieved by the RA model whilst using less memory and learnable parameters and/or learning iteration steps.

#### 3.3.3. Experiment two

The second type of run tests the management of interference between new and prior learning.

In the RA model, instances of categories 1-15 are presented first and the basal ganglia gets correct category identifications to guide acquisition of recommendation weights for those categories (i.e., consequence feedback). Then the other 15 categories are presented, and the basal ganglia gets correct category identifications for those categories. Then all 30 categories are presented and the effect of new learning on prior learning is determined.

Once again, second and third choices are also determined. Immediately after learning the second set of categories, first choice identification accuracy for the first learned set drops to about 57%, although the total for first, second and third identifications of this first learned set is close to 76%. So although accuracy has gone down, significant information has been retained.

After this, weights are adjusted in response to simple correct/incorrect feedback without identifying the correct category with another set of 10 instances of each category. Accuracy is then about 62% for first choices of both early and later categories, and 80% for combined first, second and third choices. In other words, accuracy is about the same for both the early and later categories, although somewhat lower than when all categories are introduced at the same time.

Likewise in the SOTA AI models, the first 15 categories are presented during training. Then, the next 15 are presented for training. Testing then occurs on all 30 categories and accuracy measures are recorded.

Since only correct/incorrect feedback can not be given in backpropagation models (backpropagation relies on a measure of error to update), an extension of this run type should have been performed with the E-SARSA model, which can give correct/incorrect feedback in the form reward. Although, the task appears to be too hard for a sparse E-SARSA reward model.

We designed this experiment to show how new learning degrades prior learning. The second run in this experiment type is used to show how only reward based consequence feedback affects learning. A third run is conducted as a control that uses a single pass of 30 iterations with normal consequence feedback learning to determine if the effects of correct/incorrect feedback are due to more learning iterations. We would expect to see the RA retains better accuracy on all 30 categories after split training if restricting consequence feedback outside of condition definition is a successful learning method with fewer resources. We then expect that this result carries over to types of consequence feedback that are reward based only, as in the case of the RA with a single correct/incorrect learning pass method.

## 4. Results

### 4.1. Experiment one

In Table 1, we show the resources needed to get the RA and AI models to 77% accuracy, which involves the training computations needed to correctly identify 23 out of the 30 categories presented during testing. This table shows that the RA model can learn with less resources. This is due to the ability of the cortex in the RA to define new conditions quickly by slightly modifying recommendation behavior of the condition definitions throughout learning. Parameter computation over all iterations is 477 times less in the RA model when compared to DQN (the most resource intensive protocol). Notably we see that the RA model outperforms DQN and ResNet in the number of learning parameters, training iterations needed, and the MB of RAM to conduct learning.

**Table 1:** Seventy-seven percent accuracies.

**77% Accuracy for category identification in test**			
**Model**	**RA**	**DQN**	**ResNet**
Learnable parameters	195,450	4,665,660	11,191,902
Training iterations	900	18,000	1,500
Parameters X iterations	175,905,000	83,981,880,000	16,787,853,000
MB of RAM	200	3,663	2,116

Resource metrics used during training for achieving 77% accuracy on category identification during testing.

These findings are consistent with the expectation that consequence feedback, either error or reward, degrades the ability to learn novelty efficiently because nodes have to be continuously re-tuned, not learned “on-the-fly” (as in the case of the RA model). Whereas, the RA model is trained to 77% accuracy by the 10th iteration of the x-axis, the AI models are just beginning to learn.

With 15 columns, the RA model gets about 77% correct category identifications. An additional point is that because recommendation strengths are available for every category, it is possible to calculate second and third choices etc. of cortical outputs. Cognitively this would correspond with identifying a category, getting immediate feedback that the choice was wrong, and selecting the next highest recommendation strength. The percent in which either the first or the second choice is correct is about 90%, and about 95% for first, second or third.

When the number of columns is increased to 20, correct first choices are 84%, first or second 94%, and first or second or third 97%. For a cortex with 20 columns, increasing the number of presentations to 1,500, with basal ganglia learning occurring in an extra 300 results in an increase in correct choices for first, first or second, and first second or third to 87, 95, and 98% respectively. A run was also performed with 20 cortical columns and 1,500 presentations, and with changes to the connectivity of layer one in each column. The number of layer one neurons was increased, the number of dendritic branches on each layer one neuron was increased, and the number of inputs to each branch. In this run, correct first choices increased to 94%, first or second to 99% and first or second or third to 100%.

The RA model was configured to increase accuracy on the task to 90% (see Supplementary Section 4 for exact configuration). Results are shown in comparison with the SOTA AI models in Table 2.

**Table 2:** Ninety-four percent accuracies.

**90% Accuracy for category identification in test**			
**Model**	**RA**	**DQN**	**ResNet**
Learnable parameters	460,600	4,665,660	11,191,902
Training iterations	1,200	27,000	3,000
Parameters X iterations	552,000,000	125,972,820,000	33,575,706,000
MB of RAM	320	3,663	2,119

Resource metrics used during training for achieving 94% accuracy on category identification during testing.

These findings are consistent with previous findings that the RA model is more efficient, but also establishes that overall performance (i.e., maximum accuracy) is not diminished. All models are capable of reaching at least 90% accuracy. It is likely that the RA model could achieve increased accuracy, however this work will be implemented in future experimentation on accelerators. Yet, the orders of magnitude difference between resource consumption indicates the RA model would likely still out-perform the SOTA AI models in terms of resource efficiency per accuracy performance.

### 4.2. Experiment two

Table 3 compares approaches of retaining learning in the categorical task when models were trained to approximately 77% accuracy.

**Table 3:** Accuracy percent during split training.

**Accuracy for category identification in test split training**			
**task**			
**Model**	**RA**	**DQN**	**ResNet**
Normal training	57.6%	31.5%	44.3%
Correct/Incorrect	62.1%	n/a	n/a
Full training	78.6%	47%	27.7%
MB of RAM	320	3,663	2,119

Training accuracies on test phase across models and types of split task learning protocols.

We see that learning is slightly diminished in the RA model when returning to the task (77% accuracy to 57.6% accuracy). In the SOTA models, an even larger decrease in accuracy is observed after learning the second set of category tasks. This is likely because knowledge retention is preserved better in the RA model, which is expected since receptive fields in the cortical module are not being re-written for the sake of the second set of categories during learning.

Notably, the drop in knowledge retention in the RA model can be largely recovered with one more pass through all 30 categories with a simple correct or incorrect feedback, shown in Table 3 (62.1%).

**Figure 3:**
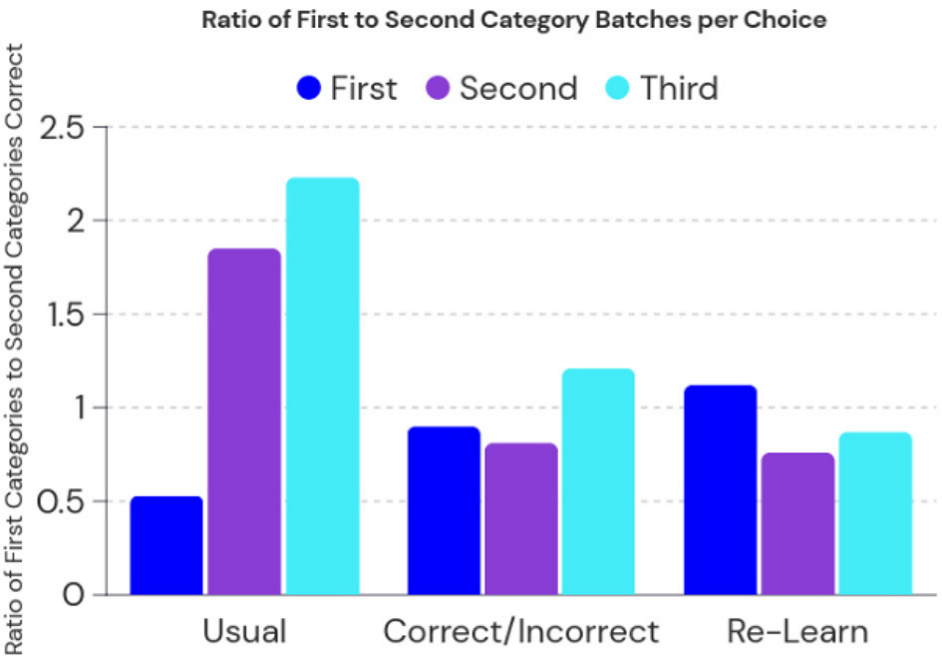
The RA model performance on the split learning task. Usual indicates no further learning was used after the split task, correct/incorrect indicates feedback was given in a single pass extra learning after split task, and re-learn indicates full re-learning of all 30 categories. A ratio close to one would mean a balanced learning of both first and second sets of categories.

To better understand the ability of the RA model to preserve knowledge, we evaluate the ratio between the first and second category learning sets during testing. The resulting Figure 3 shows the direction of bias on preserved knowledge while comparing the original split learning task, the correct/incorrect split learning tasks, and a control of completely re-learning all 30 tasks after the split learning procedure. First, second, and third choices correspond to the output layer in the basal ganglia in which the first guess is usually taken as the accuracy measure. The second and third are provided as an indication if the model was initially incorrect, how would it be close to identifying the correct category.

We then compare each learning method in the split task, we see that returning to learning does have a bias on the second set of categories. However, using another learning pass of only correct/incorrect feedback not only recovers accuracy but also establishes a ratio close to one. This indicates that there is a balanced retention of knowledge between the first set of categories and second set of categories. Interestingly, we see that completely re-learning all categories has a slight bias toward the first set of categories, this is likely because some of the weights are over-reactive toward the first set of categories in an attempt to recover the imbalance during split training.

## 5. Discussion

In this work, we show that by restricting consequence feedback learning to interpretation of cortical patterns of activation to recommend behaviors, and while cortical patterns of activation are managed by synaptic connectivity and weight changes based on frequency of inputs often occurring together in the past, learning steps and number of learnable parameters can be reduced massively while still retaining performance in learning continuously new inputs. We further show that new learning does not completely degrade prior learning within this framework.

Through manual hyperparameter tuning in the RA model, we discover accuracy increases with the number of columns. Accuracy is also considerably increased by the bias on initial connectivity to columns in favor of different groups of inputs that have often occurred at the same time in past (otherwise unprocessed) category instances. Accuracy is also sensitive to the general reduction in synaptic strengths of neurons that fire very often. See Supplementary Section 6 for details.

From a consciousness approach, this work sheds light on the discussion of the neural basis of learning in cortical and subcortical brain areas. Results comment on the tendency to associate cortical activation with consciousness, in which we show here can be based on learning not entirely consequence feedback driven. Specifically, error and reward are not the only viable interpretations of learning in the brain and when they are treated as such, knowledge retention is preserved.

Works from Torbert et al. (1972), Sun (1997), Coward (1999b), Cleeremans and Jiménez (2002), and Cleeremans and Frith (2003) have attempted to argue that experiential learning proliferates consciousness (or vice versa), while others Lewicki et al. (1992), Phaf and Wolters (1997), Hobson and Pace-Schott (2002), Olcese et al. (2018), and Birch et al. (2020) have come from the angle of studying learning in conscious and nonconscious forms. Results here, constructed from biologically supported mechanisms, provide insight into learning that is conscious and nonconscious in an integrated model.

We provide a model to start testing underlying mechanisms of consciousness as proposed in several prior works (Cleeremans, 2005; Seth, 2009). Promising directions involve modeling theories of consciousness, such as the RA consciousness framework and Global Workspace within the RA model framework. Appropriate modifications to the model would allow a more biologically plausible evaluation environment than that of deep learning models, in regards to cognition and consciousness studies.

Furthermore, the basal ganglia and hippocampus modules are severely underdeveloped. Future work would benefit from extending these modules to more realistic instantiations based on neuroscience literature for various sleep and behavior learning studies.

This paper also contributes to the field of artificial intelligence in the hopes that as AI models continue to dominate practical applications and become state-of-the-art tools in brain modeling, more biologically plausible and scalable (i.e., resource efficient) architectures can be adopted. Further, some of the findings here support that backpropagation and reinforcement learning are not suitable for general learning as it occurs in humans because consequence feedback degrades learning stored in condition definitions. We believe this paper provides counter-evidence to the emerging trend that more computations lead to greater intelligence.

To more accurately describe the relationship between consequence feedback and condition definition in learning models, the RA model needs to be ported to accelerated frameworks (e.g., pytorch) such that it can be more rigorously compared to existing models in AI. One limitation of the study is that since the RA model is written in the Smalltalk programming language and AI models are typically written in Python with GPU acceleration, collecting a time analysis was not feasible. Future work will focus on creating a model of RA that fits into the existing and predominant AI development tools.

## 6. Conclusion

It is our aim that this discussion encourages future work on the relationship between consequence feedback, learning, and consciousness. We've shown that learning can occur within resource constraints (i.e., several orders of magnitude fewer resources) when condition definition (i.e., features defined by cortical neurons) are learned through plasticity and Hebbian principles, not reward or error.

Subsequent work could provide insight into how each brain region (i.e., basal ganglia, hippocampus, etc.) affects learning and to further explain how theories of consciousness relate to learning within this framework. Other studies are needed to compare the RA model to other cognitive architectures rather than deep learning architectures alone.

## Data availability statement

The datasets presented in this study can be found in online repositories. The names of the repository/repositories and accession number(s) can be found below: The datasets generated for this study can be found in the github repository Novel Category Task. https://github.com/simuliinc/Novel-Category-Task.

## Author contributions

RS and LC contributed to conception and design of the study and wrote the first draft of the manuscript. SS organized the discourse. RS performed the experiment execution and analysis relating to SOTA AI models. LC performed the experiment execution and analysis relating to the RA model. All authors contributed to manuscript revision, read, and approved the submitted version.
