# Supplementary material for: Leveraging conscious and nonconscious learning for efficient AI
Source: Front Comput Neurosci. 2023 Mar 23;17:1090126. doi: 10.3389/fncom.2023.1090126 (PMC10076654; doi:10.3389/fncom.2023.1090126)

1 DQN

a.

Hyperparameter tuning parameter range						
Gamma	0.999	0.6	0.3	-	-	-
Learning Rate	0.001	0.0001	0.0005	-	-	-
Target Update	5	10	20	-	-	-
Number of Episodes	300	500	700	-	-	-
Batch Size	32	64	128	256	512	-
Epsilon Decay	100	200	300	-	-	-
Epsilon End	0.01	0.05	0.1	-	-	-
Epsilon Start	0.99	0.9	0.7	-	-	-
Layer One Nodes	5	10	16	20	32	64
Layer Two Nodes	10	20	32	64	128	-

b.

Selected hyperparameters after 13 model runs	
Gamma	0.999
Learning Rate	0.0001
Target Update	10
Number of Episodes	900
Batch Size	32
Epsilon Decay	200
Epsilon End	0.01
Epsilon Start	0.99
Layer One Nodes	32
Layer Two Nodes	128

c.

Model setup	
Layer One	2D convolutional layer, kernel size of 3, stride of 2
Dropout	2D Batch Norm
Activation	ReLU
Layer Two	2D convolutional layer, kernel size of 3, stride of 2
Dropout	2D Batch Norm
Activation	ReLU
Layer Two	2D convolutional layer, kernel size of 3, stride of 2
Dropout	2D Batch Norm
Activation	ReLU
Output Layer	fully connected size 30

2 ResNET

a.

Hyperparameter tuning parameter range				
Number of Episodes	50	100	200	300
Learning Rate	0.001	0.0001	0.0005	-
Layer One Nodes	ResNet 18	ResNet 34	ResNet 50	ResNet 101

b.

Selected hyperparameters after 13 model runs	
Number of Episodes	100
Learning Rate	0.0001
Layer One Nodes	ResNet 18

c.

Model setup	
All Layers	resnet18
Output Layer	fully connected size 30

3 E-SARSA

a.

Hyperparameter tuning parameter range	
Alpha	
Epsilon Start	
Epsilon End	
Epssilon Decay	
Gamma	
Number of Episodes	

b.

Selected hyperparameters after 13 model runs	
Alpha	
Epsilon Start	
Epsilon End	
Epssilon Decay	
Gamma	
Number of Episodes	

4 RA

a.

Model parameter configuration		
Accuracy on Test	77.40%	89.80%
Columns	15	20
Layer one neurons	10	20
Branches per layer one neuron	50	50
Inputs per layer one neuron branch	20	20
Layer two neurons	10	10
Branches per layer two neuron	10	10
Inputs per layer two neuron branch	15	15
Layer three neurons	10	10
Branches per layer three neuron	10	10
Inputs per layer three neuron branch	15	15
Cortical synapses	195000	460000
Basal Ganglia synapses	450	600
Total synapses	195450	460600

b.

Split learning model parameter configuration	
Columns	15
Layer one neurons	10
Branches per layer one neuron	50
Inputs per layer one neuron branch	20
Layer two neurons	10
Branches per layer two neuron	10
Inputs per layer two neuron branch	15

Layer three neurons	1
Branches per layer three neuron	10
Inputs per layer three neuron branch	15
Cortical synapses	174750
Basal Ganglia synapses	450
Total synapses	175200
Number of Learning Episodes	1650

5 DQN with working memory training accuracy for only 6 categories for four different runs. This model failed to learn.

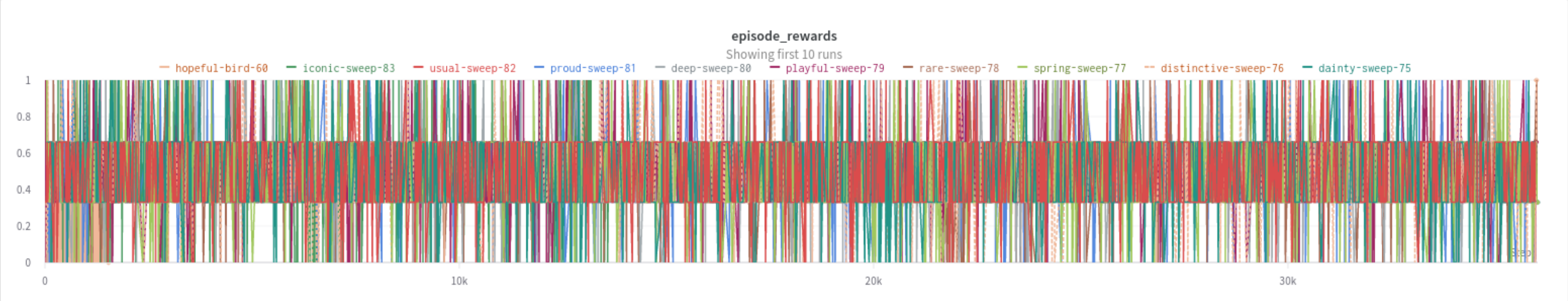

6 RA results on 200 category identification tasks, with varied recommendation weights.

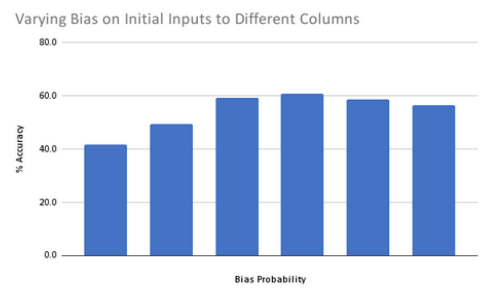

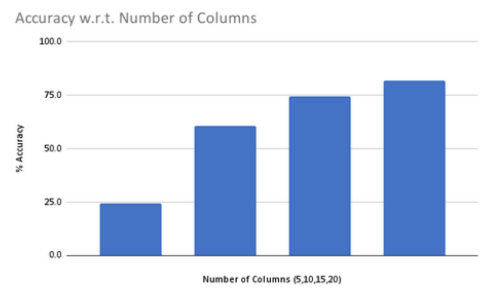

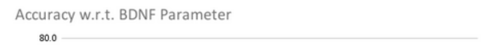

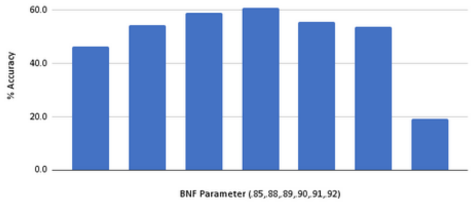

Accuracy w.r.t. Weight Reduction Factor

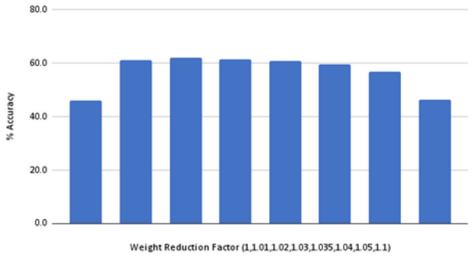

Supplement: Supplementary file 1 [file Data_Sheet_1.PDF]
